# The Role of Human Parietal Area 7A as a Link between Sequencing in Hand Actions and in Overt Speech Production

**DOI:** 10.3389/fpsyg.2012.00534

**Published:** 2012-12-05

**Authors:** Stefan Heim, Katrin Amunts, Tanja Hensel, Marion Grande, Walter Huber, Ferdinand Binkofski, Simon B. Eickhoff

**Affiliations:** ^1^Section Structural Functional Brain Mapping, Department of Psychiatry, Psychotherapy, and Psychosomatics, Medical School, RWTH Aachen UniversityAachen, Germany; ^2^Research Centre Jülich, Institute of Neuroscience and Medicine (INM-1)Jülich, Germany; ^3^Section Clinical and Cognitive Neurosciences, Department of Neurology, Medical School, RWTH Aachen UniversityAachen, Germany; ^4^JARA – Translational Brain MedicineJülich and Aachen, Germany; ^5^Institute of Clinical Neuroscience and Medical Psychology, Heinrich-Heine University DüsseldorfDüsseldorf, Germany

**Keywords:** language, speech, aphasia, fMRI, broca, apraxia, repetition

## Abstract

Research on the evolutionary basis of the human language faculty has proposed the mirror neuron system as a link between motor processing and speech development. Consequently, most work has focused on the left inferior frontal cortex, in particular Broca’s region, and the left inferior parietal cortex. However, the direct link between planning of hand motor and speech actions has yet to be elucidated. Thus, the present study investigated whether motor sequencing of hand vs. speech actions has a common neural denominator. For the hand motor task, 25 subjects performed single, repeated, or sequenced button presses with either the left or right hand. The speech task was in analogy; the same subjects produced the syllable “po” once or repeatedly, or a sequence of different syllables (“po-pi-po”). Speech motor vs. hand motor effectors resulted in increased perisylvian activation including Broca’s region (left area 44 and areas medially adjacent to left area 45). In contrast, common activation for sequenced vs. repeated production of button presses and syllables revealed the effector-independent involvement of left area 7A in the superior parietal lobule (SPL) in sequencing. These data demonstrate that sequencing of vocal gestures, an important precondition for ordered utterances and ultimately human speech, shares area 7A, rather than inferior parietal regions, as a common cortical module with hand motor sequencing. Interestingly, area 7A has previously also been shown to be involved in the observation of hand and non-hand actions. In combination with the literature, the present data thus suggest a distinction between area 44, which is specifically recruited for (cognitive aspects of) speech, and SPL area 7A for general aspects of motor sequencing. In sum, the study demonstrates a previously underspecified role of the SPL in the origins of speech, and may be discussed in the light of embodiment of speech and language in the motor system.

## Introduction

“Every journey begins with a first step” – This Chinese proverb poetically characterizes a process most relevant in many domains of human life: sequencing. Sequencing means that complex actions such as speaking or grasping are broken down into a series of individual steps or sub-processes, which are taken in a particular order as time unfolds. As we grasp a key to unlock a door, we perform a series of arm, hand, and finger movements targeted toward the key, to move it toward the door lock, and to turn it. As we speak, we create a syntactic sentence structure in our mind, fill its slots with words, serially retrieve the words’ phonemes and realize them sequentially with our articulators as series of vocal gestures.

The outstanding question is therefore whether “sequencing” is one common process which supports the domains of motor actions and speech production alike, or whether each domain has its own sequencing affordances and modules. About 40 years ago, Doreen Kimura addressed this question in a number of well-designed and, at that time, technically well advanced studies (e.g., Kimura, 1973; Lomas and Kimura, 1976; see also Kimura, 1993). Kimura (1973) investigated whether hand motor actions in right-handers would occur during speaking, and if so, more frequently than during humming. Indeed, this was what she found, concluding that speaking as a mouth motor act was closely linked to other, non-mouth, motor acts. Together with Jonathan Lomas, she followed up on this experiment (Lomas and Kimura, 1976), this time investigating the reverse logic: would the deliberate execution of finger tapping sequences be affected by simultaneous speech acts? The authors did observe this finding, which was reported, again, for right-handers but which was likewise found for left-handers. Interestingly, for the purpose of the present argument, the study did explicitly investigate sequences of finger movements, which were distinguished by the authors as complex movements (though members from the same limb, i.e., the arm) from mere repetitious finger tapping. Together, Kimura makes an explicit statement that sequences of finger movements are certainly related to articulatory gestures constituting mouth muscle movements. On the basis of 20 years of accumulated evidence, Kimura (1993) discusses that this shared movement control is likely linked to the parietal lobe, which is supposed to be in charge for finger movements as well as for articulatory movements. However, there is also more recent, contradictory evidence (e.g., Maas et al., 2008) that finger movements and speech movements are differentially affected by the length of a produced sequence, which may be a hint at a partial dissociation of these effector domains with respect to sequencing.

A more recent analysis of cognitive and neural components commonly involved in speech and praxis indeed suggests that both domains rely on shared resources (Roby-Brami et al., 2012). These hypothetically shared resources involve the “nesting of chunks and sequences,” which may happen when complex hierarchies are broken down into sequences (Koechlin and Jubault, 2006). In fact, the model by Craighero et al. (2007) which links moves, acts, and actions as different hierarchical levels of movements very much resembles linguistic hierarchies, such as syntactic tree structures.

The concept of “sequencing,” however, may actually comprise several aspects, in particular one “cognitive,” i.e., the mental planning of a sequence, and one “motor” focusing on the actual serial execution of units or chunks of actions. Following the two-stage model by Klapp (1995), Maas et al. (2008) discussed and investigated this issue in patients with apraxia of speech. In this sample, the cognitive “preprogramming” stage could be clearly dissociated from the subsequent process of ordering the elements for and during execution.

Several groups have approached the issue of shared sequencing resources in motor praxis and speech with functional neuroimaging studies on healthy volunteers. Reviewing the available evidence, Fiebach and Schubotz (2006) hypothesized that a promising candidate region could comprise Broca’s speech region in the left inferior frontal cortex plus the caudally adjacent ventral premotor cortex. This hypothesis was based on the observations that (1) phonological sequencing relies on the posterior-superior aspect of the left inferior frontal gyrus and the precentral gyrus (e.g., Demonet et al., 1992; Zatorre et al., 1996), (2) syntactic sequencing involves the posterior-inferior portion of the left inferior frontal gyrus and the foot of the precentral gyrus (e.g., Indefrey et al., 2001; Grewe et al., 2005), (3) serial prediction of events recruits the premotor cortex (Schubotz and von Cramon, 2002), and (4) a lesion in this region hampers serial predictions (Schubotz and von Cramon, 2004). Furthermore, from today’s perspective, this proposal is also in line with the observation of a gradient in Broca’s region from hierarchical (anterior IFG) to sequence (posterior IFG and premotor cortex) processing (Koechlin and Jubault, 2006).

However, there are a couple of potential arguments against the idea of a shared neural sequencing module in motor actions and speech that should be considered, one neurofunctional and one neuroanatomical. First, neurofunctionally, there is growing consensus that higher cognitive functions such as sequencing are supported by distributed networks, rather than isolated functionally specified regions (for the phonology-to-articulation loop cf. Eickhoff et al., 2009). In particular, both speech processing (involving phonological sequencing) and motor sequencing additionally recruit the several aspects of the parietal cortices (motor: e.g., Jeannerod et al., 1995; Haslinger et al., 2002; Rumiati et al., 2004; speech: see Vigneau et al., 2006, for a review). Thus, it is not necessarily the case that one neuroanatomical module such as “Broca’s region” would support sequencing; rather, a set of regions could be involved. Second, from clinical studies it is known that learning of motor sequences and of phonemic sequences differs in Broca’s aphasia: whereas such patients, who had left frontal lesions, generally performed normally in motor sequence tasks, they were selectively impaired in learning phoneme sequences (Goschke et al., 2001; However, note that training of non-phonological sequences may improve syntactic abilities in these patients: Hoen et al., 2003, and that TMS to area 44 also impairs motor sequence learning: Clerget et al., 2012). Finally, from a neuroanatomical point of view, cytoarchitectonic and receptorarchitectonic mapping of the human cerebral cortex since Brodmann’s (1909) early work allows an increasingly fine-grained description of brain regions within the “premotor” or “prefrontal” cortex which differ substantially in their microarchitecture – and consequently most likely also in their function(s). Amunts et al. (2010) demonstrated that Brodmann’s areas 44 and 45 within Broca’s speech region each consist of two sub-regions (areas 45a and 45p; areas 44d and 44v). Likewise, the ventral premotor cortex (area 6) can be parcellated into three areas (6r1, 6v1, and 6v2). A hierarchical cluster analysis (Amunts et al., 2010) revealed a closer relationship between total area 6 with areas 44 and 45 than with total primary motor area 4, but still, they are distinct. Interestingly, not only the classical “Broca’s region,” but also the junction region of the inferior frontal sulcus and precentral sulcus which links the precentral gyrus to the inferior frontal gyrus is architectonically distinct (areas ifj1 and ifj2). The same argument holds for the “parietal cortex” with eight superior parietal areas (5Ci, 5M, 5L, 7PC, 7A, 7P, 7M, and hIP3; Scheperjans et al., 2008) and seven inferior parietal areas (PGa, PGp, PFop, PFt, PF, PFm, and PFcm; Caspers et al., 2006) which are separated by yet distinct areas (hIP1, hIP2; Choi et al., 2006) in the intraparietal sulcus. Thus, even though, macro anatomically, neighboring regions are involved in sequencing in the speech and in the hand motor domain, it remains to be shown that these also involve the same cytoarchitectonically defined areas.

To summarize, the current view on “sequencing” regions in the motor and speech domains appears as follows. (1) At a cognitive level, sequencing may be a component that is shared, as module, in different variants of motor and speech processing. Given the heterogeneous findings listed above, a distinction between “cognitive sequencing” (involving a planning stage) and “motor sequencing” (involving the ordered execution), and consequent focus on the one or other, might further clarify matters. (2) At a neurofunctional level, there are hints at potentially shared neural systems in frontal and parietal regions. (3) At a neuroanatomical level, it is far from clear whether such systems recruit the same, or rather neighboring, cytoarchitectonic areas.

The present study was therefore designed to investigate motor sequencing in finger movements and in speech production in healthy controls using functional magnetic resonance imaging (fMRI) in combination with the Düsseldorf-Jülich cytoarchitectonic brain atlas.

In particular, we addressed the following question: are there brain regions involved in sequencing that are shared in finger movements and speech production?

## Materials and Methods

### Participants

Twenty-five healthy volunteers (average age: 35 years; age range 30–61 years; 11 women) with no reported history of neurological or psychiatric disorders participated in the study. All were right-handed according to the Edinburgh Handedness Inventory (Oldfield, 1971) and received 15 EUR for their participation. Informed consent was obtained from all participants. The study was approved by the Ethics Committee of the Medical Faculty of RWTH Aachen University.

### Procedure

The functional scan consisted of six blocks, two for the right hand (R), two for the left hand (L), and two for overt speech (S). In each block, the participants completed trials for three different modes which were presented in a pseudo-randomized order: trials with a single response (ONE), trials in which the same response was repeated three times (REP), and trials in which a sequence of different responses was performed (SEQ). Combining three response modes (right hand, left hand, speech) with three gave a total of nine experimental conditions (R-ONE, R-REP, R-SEQ, L-ONE, L-REP, L-SEQ, S-ONE, S-REP, S-SEQ). A visual cue at the beginning of each trial indicated which response was required. In R-ONE trials, the participants pressed a response button once with the index finger of the right hand. For R-REP, they pressed the same button three times repeatedly. Finally, for R-SEQ, they pressed an alternating sequence with their right index, middle, and index finger. The same was done with the index finger (or index and middle fingers) of left hand for L-ONE, L-REP, and L-SEQ. Finally, for spoken responses, S-ONE required saying the syllable “po,” S-REP saying the same three times (“po-po-po”), and S-SEQ producing a diadochokinesis-like sequence “po-pi-po.” In each of the six blocks, there were 30 trials for each response mode (ONE, REP, SEQ) plus 10 null events, amounting to a total of 540 task events completed by each participant.

Each trial started with an empty screen for 1900 ms, followed by the presentation of a fixation cross for 1000 ms. Then, the visual task cue appeared for 1000 ms, and the participants gave their (hand motor or spoken) response while the task cue remained on the screen for the rest of the trial. In the null events, a blank was shown instead of the cue. Responses were restricted to a time window of 1000 ms (see below). Practice trials before scanning ensured that the subjects were familiar with all conditions and responded correctly and with constant speed to the cue.

### Data acquisition

Scanning was performed on a Trio 3T scanner (Siemens, Erlangen, Germany) located at the Research Centre in Jülich. A time series of 616 T2*-weighted EPI images (flip angle FA: 90°; echo time TE: 30 ms; field of view FOV: 200 mm; matrix: 64 × 64) were acquired from 49 sagittal slices with a thickness of 3 mm. The sagittal orientation of the slices was chosen in order to better correct for nodding head movements in the *y*-*z*-plane (pitch rotation; Heim et al., 2006). Moreover, in order to dissociate BOLD acquisition from overt speaking, a sparse-sampling sequence was used with a repetition time (TR) of 4000 ms and a delay-in-TR of 1000 ms at the end of each TR (Heim et al., 2006). Using this procedure, all data were recorded during the first 3000 ms in each TR. During the last 1000 ms when the task cue occurred and the motor reaction (button presses or speaking) was performed, no data were acquired. By this, motion and data recording are dissociated and motion-induced artifacts in the BOLD signal can be avoided.

After the functional scan, a T1-weighted MPRAGE sequence (FOV 256 mm; TR 2250 ms; TE 3,03 ms; FA: 9°) was run in order to obtain structural images of each participant’s brain at an isotropic resolution of 1 mm × 1 mm × 1 mm.

### Data analysis

Analysis of the functional data was performed with SPM5 (Wellcome Trust Centre for Neuroimaging, UK) running on Matlab 7.0 (The MathWorks, Inc., USA). Images were first corrected for head movement by affine registration using a two-pass procedure, by which images were initially realigned to the first image and subsequently to the mean of the realigned images. After realignment, the mean EPI image for each subject was spatially normalized using the “unified segmentation” approach (Ashburner and Friston, 2005). The resulting parameters of a discrete cosine transform, which defined the deformation field necessary to warp the subjects data into the space of the Montreal Neurological Institute (MNI) tissue probability maps, were applied to the individual EPI volumes and re-sampled at 2 mm × 2 mm × 2 mm voxel size. The normalized images were spatially smoothed using an 8 mm full-width at half-maximum Gaussian kernel to meet the statistical requirements of the general linear model (GLM) and to compensate for residual inter-subject variations in brain anatomy.

The first level event-related statistical analysis of each individual data set consisted of convolving the onset vectors for each of the nine conditions with the canonical hemodynamic response function. Estimates of the regressors for each condition were obtained by contrasting each condition against the implicit resting baseline (contrasts of the type “1 0 0 0 …”). These contrast images were then entered into a random-effects group analysis at the second level with “subject” as repetition factor and “condition” as experimental factor. In this analysis, we used the following contrasts in order to identify, and juxtapose, effector-independent networks for motor sequencing vs. repetition:

a) Main effect left hand sequencing (L-SEQ > L-REP)
b) Main effect right hand sequencing (R-SEQ > R-REP)
c) Main effect speech sequencing (S-SEQ > S-REP)
d) Conjunction analysis (SPM option “conjunction null”) of the three main effects for SEQUENCING, revealing regions commonly involved in sequencing in all three effectors, i.e., independently of effector which are assigned the least of the *t* values of the three maps entered into the analysis.

All individual contrasts were calculated at *P* < 0.05 family wise error (FWE) corrected at cluster level (cluster-forming threshold at voxel-level *p* < 0.001, required extent threshold *k* ≥ 250 voxels). In a further step, the conjunction analyses were computed based on the resulting three maps for speech, left hand, and right hand.

For the anatomical localization of the activations we used cytoarchitectonic probability maps, which are based on an observer-independent analysis of the cytoarchitecture in a sample of 10 post-mortem brains (Zilles et al., 2002; Schleicher et al., 2005). They provide information about the location and variability of cortical regions in standard MNI reference space. For the assignment of MNI coordinates to the cytoarchitectonically defined regions we used the SPM Anatomy Toolbox (Eickhoff et al., 2005, 2006, 2007).

## Results

### Sequencing

**Figure 1:**
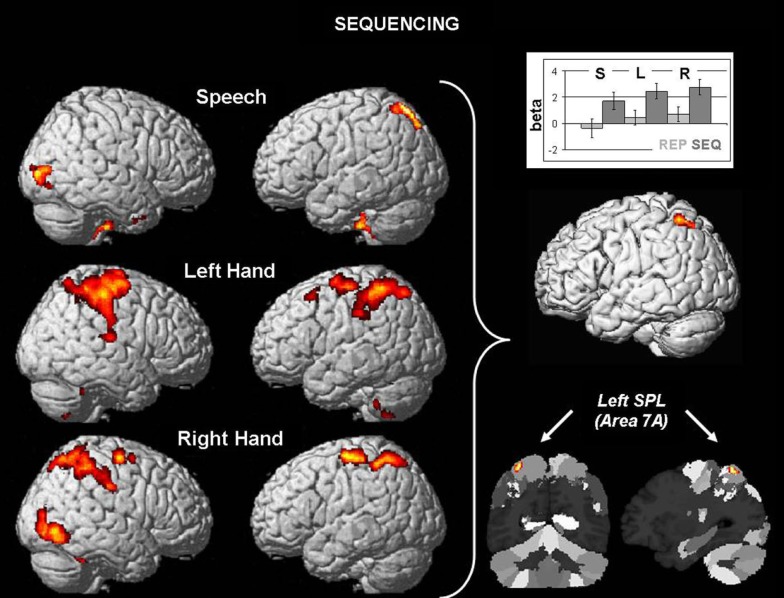
**Left: surface rendering of differential brain activation in the sequencing condition (A-B-A) as compared to repetition (A-A-A) as a function of effector (speech, left hand, right hand)**. All results are displayed at an FWE-corrected threshold of *p* < 0.05, effected as uncorrected maps at *p* < 0.001 with a cluster size of *k* ≥ 250 voxels. Right: Conjunction of the three sequencing effects yields one common region in the left superior parietal lobule in area 7A. Sections illustrate the position of the activation relative to the cytoarchitectonic maximum probability map (Scheperjans et al., 2008). The bar graphs shows the consistency of higher activation in the sequencing conditions (dark gray) as compared to repetition (light gray) in speech (S), left hand movements (L), and right hand movements (R).

The results for sequencing effects (SEQ > REP) are displayed in Figure 1. The conjunction analysis of common sequencing effects for speech production, left hand motor responses, and right hand motor responses were located in cytoarchitectonic area 7A in the left superior parietal lobule (SPL; Scheperjans et al., 2008), with an overlap of 96.8% of the cluster volume (205 voxels) with that area. The local maximum (*t* = 4.21) was located at MNI coordinates (*x*, *y*, *z*) −32, −59, 63 and assigned by the Anatomy Toolbox to area 7A. The subsequently examined beta-estimates (cf. bar graph) of each condition demonstrates the strong involvement of area 7A in all three sequencing conditions, and moreover close-to-zero effects in each condition for repetition. Furthermore, we found involvement also of the right SPL for both hand motor sequencing contrasts, which, however, was absent in the speech sequencing contrast.

Broca’s region was not involved in the sequencing in the repetition contrast (a finding that is being discussed below). Therefore, two additional analyses were computed. First, in order to investigate whether the Fiebach and Schubotz (2006) idea of a shared involvement of Broca’s region holds in the sequencing conditions *per se*, i.e., without contrasting them to other conditions, the sequencing condition for each effector (S-SEQ; L-SEQ; R-SEQ) were contrasted against the implicit resting baseline, and a conjunction analysis over these contrasts was performed. Second, in order to test the hypothesis that Broca’s region supports speech processing in general more strongly than hand motor processing (cf. Jirak et al., 2010), we additionally computed the contrast Speech (all three conditions) > Hand Motor (all six conditions).

**Figure 2:**
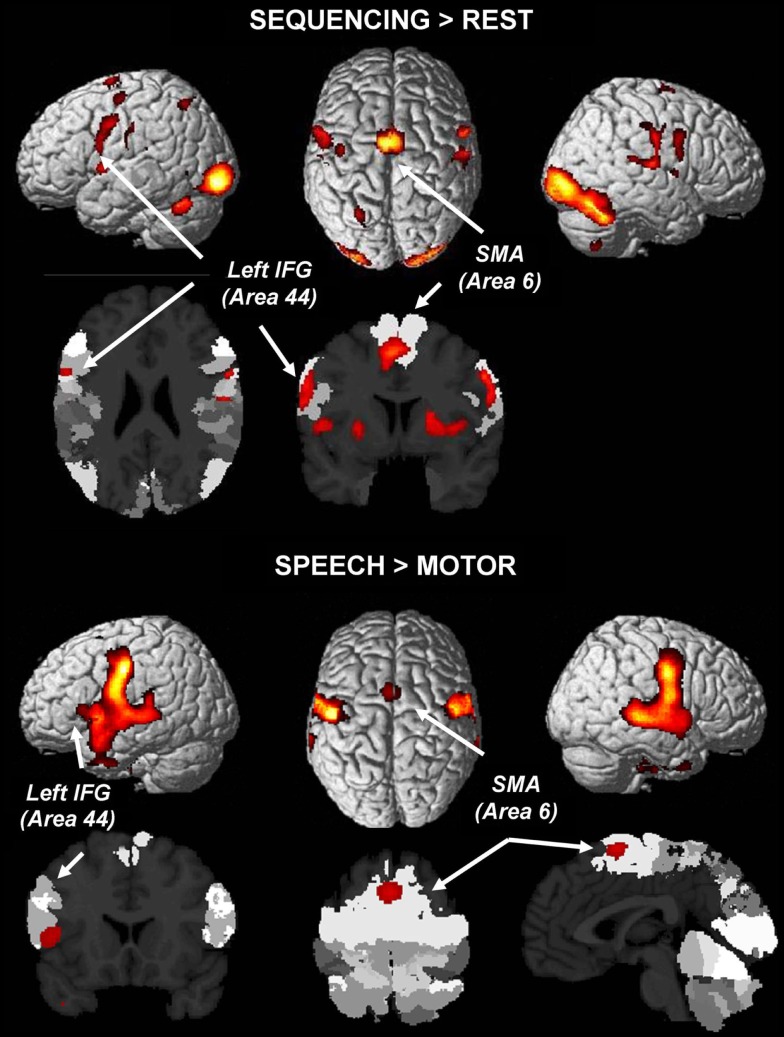
**Top: a conjunction analysis of sequencing in all three effector systems (left hand, right hand, speech) without contrasting activation against repetition (as in Figure 2) reveals largely shared networks involving left area 44 in Broca’s region (conjunction analysis of individual maps FWE-corrected at *p *< *0.05*)**. Bottom: comparing speech (single, repeated, or sequenced syllables) to button presses reveals the stronger involvement of area 44 in Broca’s region, bilateral premotor cortex, and the SMA (area 6) (FWE-corrected at *p *< *0.05*).

With respect to the first analysis, the upper part of Figure 2 shows indeed involvement of left area 44 (Amunts et al., 1999), among other regions, in the effector-independent processing of sequences. Different from the SEQ > REP analysis, this result reflects not only sequencing *per se*, but also the sensory-motor components, visual identification of the stimulus, recall of the task set, etc., i.e., components that cancel out in SEQ > REP.

With respect to Speech > Hand Motor processing, the lower part of Figure 2 reveals that this contrast indeed (again) yielded the involvement of left area 44 in Broca’s region along with an otherwise bilaterally distributed activation blob involving the SMA and covering large parts of the ventral premotor cortex and of the superior temporal cortex.

## Discussion

The present study investigated whether the process of motor sequencing is a shared resource in the speech domain and in the hand motor domain, and whether there is a common neurofunctional substrate which is distinct from brain regions distinct from brain regions merely supporting repeated movements. Indeed, by using comparisons between sequences, repetitions, or single speech or motor acts, we could identify one such region. This region was not located in the left frontal or premotor cortex, but rather in cytoarchitectonic area 7A in the SPL. This effect for sequencing was clearly distinct from that for repetitive actions, which commonly involved bilateral SMA (area 6) and bilateral IPL (areas PFcm), as well as the cerebellum.

### Motor sequencing and the left SPL (area 7A)

The one region that was significantly stronger involved in sequencing rather than repetition of right hand movements, left hand movements, and speech was area 7A in the left SPL. This shared involvement is a novel finding which, however, resonates well with previous reports on the neural correlates of sequential hand/finger movements. Van Oostende et al. (1997) could show in an early positron emission tomography study that activation in left SPL activation (presumably located in area 7 of Brodmann’s (1909) 2-dimensional map) was present when a sequence of performed finger movements had a pre-defined order, but not when fingers were moved in random order. Jenkins et al. (1994) extended these findings, showing that the SPL was in involved in both pre-learned and new sequences, but particularly when the sequences were being learned. Moreover, left SPL activation increases as a function of the number of finger orderings in tapping sequences (Boecker et al., 1998). These findings were replicated by Harrington et al. (2000), who additionally reported the importance of both the number of fingers involved in a sequence and the number of transitions between sequences. Likewise, increasing complexity of finger movements parametrically increases activation in SPL area 7 (Haslinger et al., 2002). In fact, sequencing does not have to be actually performed – mere imagery of sequencing suffices to activate the same part of the SPL as real sequences of movements (Hanakawa et al., 2008). These findings corroborate the earlier claim by Kimura (1993) of shared parietal involvement in hand and mouth motor control.

The current observation that the left SPL is involved in sequencing across modalities across modalities is thus well in line with its previously presumed (more specific) role in sequencing hand motor actions. Its left-lateralization shows that it is not related to secondary sensory-motor processing, which would depend on the use of left vs. right hand and thus recruit right and left regions, respectively. The stronger involvement of the left SPL (as compared to right SPL) within an otherwise bilateral motor network has recently been demonstrated by Otten et al. (2012), underscoring the importance of this region for abstract representations of sequencing.

At this point, it should be noted that, in general, the role of SPL area 7A is distinct from that of its neighboring area 5. Whereas area 7A has been shown to be involved in sequencing in the hand motor domain, and in the present study also in the speech domain, the function of area 5 is best defined in the context of reaching gestures in the hand motor domain. For instance, McGuire and Sabes (2011) demonstrated the involvement of area 5 when subjects moved their hands toward a target object. This differential characterization of SPL areas 7A and 5 refers back to the notion that macroanatomical labels such as “SPL” are apparently too coarse to describe the functional role of cortex areas in cognitive or motor contexts.

### Which role of Broca’s region and the left premotor cortex?

As discussed above, the finding that area 7A the left SPL plays a crucial role for sequencing also in speech nicely mirrors findings from the hand motor domain. Still, as outlined in the introduction, this result is somewhat in contrast to the prevalent hypothesis that it should be frontal, rather than parietal, areas that support sequencing. One reason can be found in the (again) rather coarse anatomical concept of a “frontal” cortex which, as discussed above, consists of several cytoarchitectonically (and presumably also functionally) distinct areas. Fiebach and Schubotz (2006) had speculated about a shared role of the premotor cortex (area 6, i.e., agranular cortex) and Broca’s region (in particular area 44, i.e., dysgranual cortex) in sequencing – or rather, cognitive sequencing as opposed to motor sequencing. The present data may contribute to distinguishing such cognitive sequencing, which has not been investigated in the present study, from actual motor sequencing tested here.

A number of studies on aspects of cognitive processing observed the involvement of Broca’s region in the processing of actions (e.g., Binkofski et al., 2000; Buccino et al., 2001; Fazio et al., 2009) – however, not necessarily in the processing of motor sequences. Jirak et al. (2010), reported activation in Broca’s region along with the SMA and premotor cortex in speech and action processing, and more strongly so for speech. This is exactly what we found in the present study (Figure 2). This comparison shows the expected involvement of area 44 and the other mentioned regions in particular for speech processing when the focus is on cognitive rather than motor sequencing.

In line with this argument that area 44 is not directly involved in motor sequencing, but may rather be involved in cognitive sequencing for all required responses (ONE; REP; SEQ), is the fact that the conjunction of all sequencing conditions (when these were contrasted only against the implicit resting baseline) again yielded a cluster overlapping with left area 44. In this contrast, cognitive sequencing, i.e., planning, does not cancel out because it is present in the task but not during rest. This may be the reason why area 44 shows up in the baseline contrast but not in the contrast explicitly tapping into motor sequencing.

### Limitations and perspectives

Having argued for a shared sequencing module in speech and hand motor processing, the question arises how generalizable this finding is – i.e., does this result extend to other fields of sequencing? As outlined above, the speech production process involves sequencing at a number of different stages, from the coordination of the articulators over the sequential retrieval and realization of phonemes and syllables to ordered words and sentences. Thus, in its most extreme form, one question would be whether complex hand and finger movements and hierarchical phrase-structure grammars (e.g., Friederici et al., 2006) have some common component. From the present findings, it would be a stretch to argue along these lines. For one, the sequences investigated here were of the type A-B-A, which does not involve much hierarchy. The use of more elaborated sequences would be required to test this assumption. The data by Harrington et al. (2000) and Haslinger et al. (2002) are promising, but much research involving comparably complex sequences in the speech and hand motor domain is needed to establish a clear neurofunctional link here.

A link that can be conceived of more easily is that to writing. Writing is an ordered finger-hand action requiring sequencing. This sequencing is based on the phoneme-to-grapheme conversion by which letters are sequentially assigned to the speech sounds of a word, and then realized as sequential hand-finger movements. Indeed, a recent study by Segal and Petrides (2012) could show the involvement of the left SPL when writing object names or copying written words was compared to naming words plus drawing loops, i.e., in a situation where motor and speech sequencing were required in coordination.

Another link worth following is that to sign language and gesturing. Indeed, sign language, like spoken language, requires substantial amounts of cognitive sequencing. Interestingly, and in contrast to spoken language, motor sequencing does not involve speech articulators but rather the hands. Thus, sign language processing represents a model in which cognitive-linguistic sequencing is linked to hand motor sequencing. In line with the present findings of the role of left SPL area 7A, a neuroimaging study by Emmorey et al. (2005) revealed the involvement of the same region, as well as of its right counterpart, during the production of American Sign Language. In a subsequent study, Emmorey et al. (2007) showed that this region was involved in both speaking and signing, with even stronger activation for signing. These findings reflect exactly the pattern of data observed for area 7A in the present study (cf. the bar graph in Figure 1). The present findings may thus contribute to a better understanding of the functional relevance of the SPL in processing sign language.

One limitation of the study is that only motor sequencing for hand and speech action was investigated, leaving the comparison to cognitive sequencing for future research. Another potential limitation could be seen in the choice of the stimuli, which consisted of sequences of one single unit (po) or two different units (po/pi), resulting in just two sequences (popopo vs. popipo). One might raise the objection that producing either popopo or popipo can be seen as producing one pre-learned item out of a set of two alternatives, with no requirements for sequencing whatsoever. Whereas it is true that a bigger set of units to be potentially combined would, from this perspective, be favorable, it might, on the other hand, have led to increased confusion of the subjects who would have had to learn more cue-response combinations in order to perform the correct response – a setting with higher cognitive demands we did not wish to include. Whether or not a larger number of stimuli would be useful, in particular to investigate cognitive sequencing, the fact remains that, at the motor level, sequencing was required for both popipo and popopo, with higher demands (due to the alternation of the units po and pi) for what we conceptualized as the “sequencing” condition. Moreover, if both popopo and popipo merely represented two templates (as instances of item learning), one would expect both conditions to elicit comparable brain activation – which was clearly not the case, in particular not in SPL area 7A. Rather, evidence from a study by Bohland and Guenther (2006) indeed suggests that it is increasing motor sequencing demands (in their case in the speech motor domain) which recruits area 7A. These authors manipulated two factors, sequence complexity (*ka-ru-ti* vs. *ta-ta-ta*) and syllabic complexity [CC(C)V vs. CV syllables, e.g., *kla-stri-splu* vs. *ka-ru-ti*] in a 2 × 2 design. While both factors yielded main effects in “BA 7” at MNI coordinates around −30, −60, 60, there was also a significant interaction: complex syllables in a complex sequence elicited higher activation than simple syllables in complex sequences or complex syllables in simple sequences. Least activation was found for simple sequences with simple syllables. This gradient reflected here is clearly a gradient of speech motor sequencing, a fact supporting the claim that it is motor sequencing that drives the activation of area 7A in the present study.

To conclude, the present study provided evidence that motor sequencing in the hand and in the speech domain shares a neural component in area 7A in the left SPL. This is a novel finding, extending data from earlier studies related only to the hand motor domain. The nature of this link needs to be investigated further with respect to hierarchies in speech sounds (e.g., complex phoneme clusters), syntax, finger movements, and complex higher-order actions not only from the motor, but also from a cognitive perspective. Moreover, the present study dissociated this sequencing finding from areas involved in repetition both in speech and finger actions, showing that sequencing of syllable production and alternating finger tapping require more than repeated access to, or execution of, movement plans. In sum, the findings are encouraging to further pursue investigations of the language-motor interface, with findings that may have implications for interventions for patients with deficits affecting one but sparing the other domain.

## Conflict of Interest Statement

The authors declare that the research was conducted in the absence of any commercial or financial relationships that could be construed as a potential conflict of interest.

## Figures and Tables

**Figure A1:**
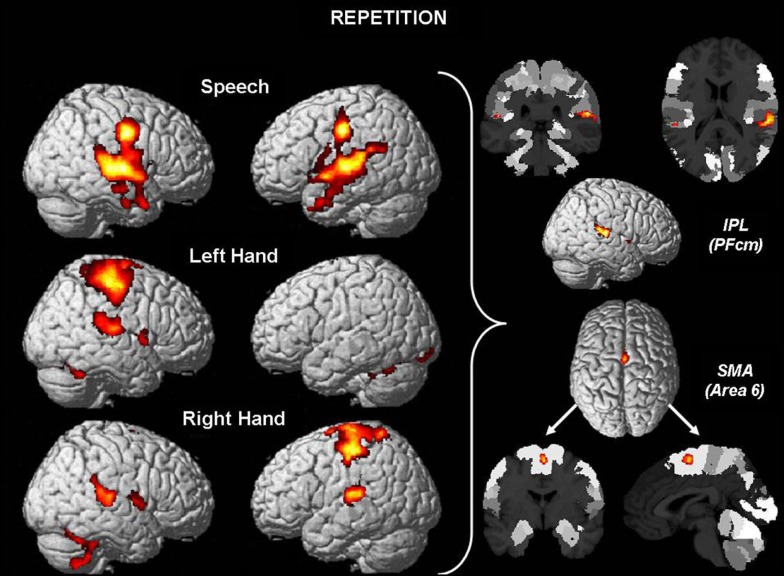
**Brain regions involved in repetition of events (three syllables, three button presses) more strongly than in single events (one button press, one syllable)**. Areas commonly involved in repetition in all three domains are area 6 in the supplementary motor area, and bilateral area PFcm in the supramarginal gyrus. For further details cf. the legend of Figure 1.

## Appendix

### Repetition

The results for repetition effects (REP > ONE) are displayed in Figure A1. The conjunction analysis of common repetition effects for speech production, left hand motor responses, and right hand motor responses yields significant effects in the left and right supplementary motor area (SMA), corresponding to area 6 (Geyer, 2003). In particular, 58.1% of the cluster volume (270 voxels) overlapped with left area 6 and 41.2% overlapped with right area 6. The local maximum (*t* = 4.11) was located in the right hemisphere at MNI coordinates 2, −3, 59.

Moreover, the inferior parietal lobule also showed shared effects of repetition in both hemispheres. One cluster was located in the right supramarginal gyrus with its local maximum (*t* = 4.66) at MNI coordinates 57, −33, 17 assigned to area PFcm. Of the total cluster volume (769 voxels), 17.3% overlapped with area PFcm, 10.5% with neighboring supramarginal area PF, and 14.6% with area OP1 in the parietal operculum. Likewise, the left homolog area PFcm was activated (97.5% of a cluster of 40 voxels), with its local maximum (*t* = 3.56) within this region at MNI coordinates −51, −33, 15. Furthermore, the right cerebellum showed a repetition effect with a cluster of 134 voxels and local maximum (*t* = 4.00) at MNI coordinates 18, −66, −20.

While the present study primarily focused on areas supporting sequencing in the speech and the hand motor domain, it was also designed to identify brain regions commonly involved in repetition of actions (as compared to single actions). Such distinction between sequencing and repetition further underscores the uniqueness of the findings for sequencing. At the same time, it enhances the understanding of the link between the speech and the hand motor system in the brain at a more general level.

In the repetition contrast, a bilateral network was observed which included areas PFcm in the supramarginal gyri and areas 6 in the left and right SMA. These findings again extend knowledge from the hand motor literature which reported the involvement of the SMA (e.g., Sadato et al., 1996; Boecker et al., 1998; Catalan et al., 1998) to the speech domain. It is tempting to interpret this finding, on a larger scale, as activation of the mirror neuron system involved not only in the execution, but also in the imagery of motion (e.g., Binkofski et al., 2000). However, a recent meta-analysis relating studies investigation action observation and execution to the cytoarchitectonic brain atlas demonstrates that it is area PFt, rather than its neighbor PFcm, which is consistently implicated in mirror neuron contexts. The function of area PFcm still remains to be characterized further in studies using the Caspers et al. (2006) maps. Given the shared involvement in speech and hand motor execution in the present study, this to-be-identified function will probably not be restricted to one domain, but to planning of repeated events independent of effector modality – but not sequencing.
